# 1-[2-(2-Bromo­phen­yl)eth­yl]-4-chloro-2-nitro­benzene

**DOI:** 10.1107/S1600536810049226

**Published:** 2010-11-30

**Authors:** Jerry P. Jasinski, Ray J. Butcher, M. S. Siddegowda, H. S. Yathirajan, A. R. Ramesha

**Affiliations:** a Department of Chemistry, Keene State College, 229 Main Street, Keene, NH 03435-2001, USA; b Department of Chemistry, Howard University, 525 College Street NW, Washington DC 20059, USA; c Department of Studies in Chemistry, University of Mysore, Manasagangotri, Mysore 570 006, India; d RL Fine Chem., Bangalore 560 064, India

## Abstract

In the title mol­ecule, C_14_H_11_BrClNO_2_, the dihedral angle between the mean planes of the bromo-substitued benzene and the chloro-substituted benzene rings is 1.8 (4) °. The nitro group is twisted by 15.8 (6)° from the mean plane of the benzene ring to which it is attached. The crystal packing is influenced by weak inter­molecular C—H⋯O inter­actions and weak π–π stacking inter­actions [centroid–centroid distances = 3.903 (2), 3.596 (2) and 3.903 (2) Å].

## Related literature

For background and a related structure, see: Post & Horn (1977 ▶). For bond-length data, see: Allen *et al.* (1987 ▶).
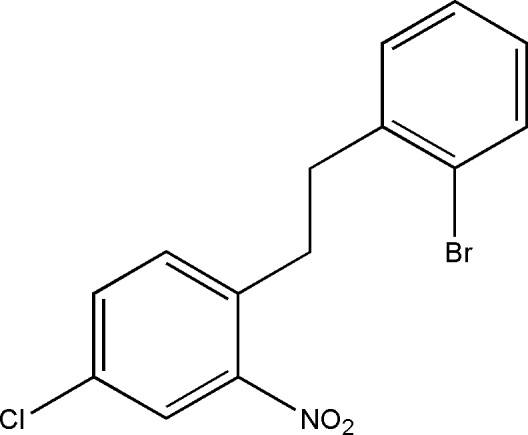


## Experimental

### 

#### Crystal data

C_14_H_11_BrClNO_2_
*M* *_r_* = 340.60
Orthorhombic, 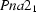

*a* = 15.7756 (4) Å
*b* = 7.3795 (2) Å
*c* = 11.5236 (3) Å
*V* = 1341.53 (6) Å^3^
*Z* = 4
Cu *K*α radiation
μ = 5.99 mm^−1^
*T* = 150 K
0.47 × 0.35 × 0.12 mm

#### Data collection

Oxford Diffraction Xcalibur Ruby Gemini diffractometer
Absorption correction: multi-scan (*CrysAlis RED*; Oxford Diffraction, 2007 ▶) *T*_min_ = 0.655, *T*_max_ = 1.000
3043 measured reflections
1901 independent reflections
1839 reflections with *I* > 2σ(*I*)
*R*_int_ = 0.021

#### Refinement

*R*[*F* ^2^ > 2σ(*F* ^2^)] = 0.031
*wR*(*F* ^2^) = 0.084
*S* = 1.08
1901 reflections
172 parameters
1 restraint
H-atom parameters constrained
Δρ_max_ = 0.51 e Å^−3^
Δρ_min_ = −0.36 e Å^−3^
Absolute structure: Flack (1983 ▶), 472 Friedel pairs
Flack parameter: 0.04 (2)

### 

Data collection: *CrysAlis PRO* (Oxford Diffraction, 2007 ▶); cell refinement: *CrysAlis PRO*; data reduction: *CrysAlis RED* (Oxford Diffraction, 2007 ▶); program(s) used to solve structure: *SHELXS97* (Sheldrick, 2008 ▶); program(s) used to refine structure: *SHELXL97* (Sheldrick, 2008 ▶); molecular graphics: *SHELXTL* (Sheldrick, 2008 ▶); software used to prepare material for publication: *SHELXTL*.

## Supplementary Material

Crystal structure: contains datablocks global, I. DOI: 10.1107/S1600536810049226/lh5167sup1.cif

Structure factors: contains datablocks I. DOI: 10.1107/S1600536810049226/lh5167Isup2.hkl

Additional supplementary materials:  crystallographic information; 3D view; checkCIF report

## Figures and Tables

**Table 1:** Hydrogen-bond geometry (Å, °)

*D*—H⋯*A*	*D*—H	H⋯*A*	*D*⋯*A*	*D*—H⋯*A*
C3—H3*A*⋯O2^i^	0.95	2.62	3.479 (6)	150
C13—H13*A*⋯O1^ii^	0.95	2.60	3.421 (4)	145

Symmetry codes: (i) 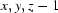; (ii) 
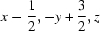
.

**Fig. 1.**
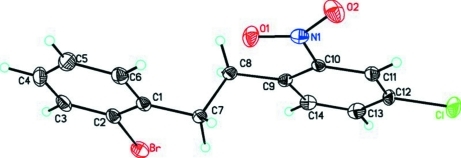
Molecular structure of the title compound showing the atom labeling scheme and 50% probability displacement ellipsoids.

**Fig. 2.**
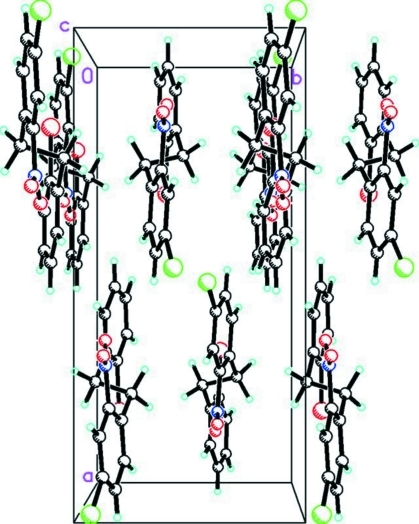
Packing diagram of the title compound viewed along the c axis.

## supplementary crystallographic information

### Comment

1-(4-Chloro-2-nitrophenethyl)-2-bromobenzene is an intermediate in the synthesis of 10,11–dihydro-5*H*-dibenzo[b,f]azepine derivatives. It has also been used successfully in the preparation of a number of biologically active compounds and drugs; *e.g.* the antidepressants imipramine, chloripramine are among the most commonly known. The crystal and molecular structure of the tricyclic antidepressant chlorimipramine hydrochloride is reported (Post & Horn, 1977). In view of the importance of the title compound as a pharmaceutical intermediate, this paper reports its crystal structure.

In the crystal structure of the title compound, C_14_H_11_BrClNO_2_, the dihedral angle between the mean planes of 1-bromo benzene and 2-nitro, 4-chloro benzene rings is 1.8 (4)Å (Fig. 1).  The nitro group is twisted 15.8 (6)° from the mean plane of the 2-nitro, 4-chloro benzene ring. Bond angles and distances (Allen *et al.*, 1987) are in normal ranges. Crystal packing is influenced by weak C—H···O intermolecular interactions (Table 2) and weak π–π stacking interactions (Fig. 2).

### Experimental

1-[2(2-Bromo-phenylethyl]-4-chloro-2-nitrobenzene was obtained as a gift sample from RL Fine Chem, Bangalore. The compound was recrystallized from dichloromethane (m.p.: 361–363 K).

### Refinement

All of the H atoms were placed in their calculated positions and then refined using the riding model with Atom—H lengths of 0.95Å (CH) and 0.99Å (CH_2_). Isotropic displacement parameters for these atoms were set to 1.18–1.21 (CH), or 1.18 (CH_2_) times *U*_eq_ of the parent atom.

### Crystal data

**Table:**

C_14_H_11_BrClNO_2_	*F*(000) = 680
*M**_r_* = 340.60	*D*_x_ = 1.686 Mg m^−^^3^
Orthorhombic, *P**n**a*2_1_	Cu *K*α radiation, λ = 1.54184 Å
Hall symbol: P 2c -2n	Cell parameters from 2576 reflections
*a* = 15.7756 (4) Å	θ = 4.8–73.8°
*b* = 7.3795 (2) Å	µ = 5.99 mm^−^^1^
*c* = 11.5236 (3) Å	*T* = 150 K
*V* = 1341.53 (6) Å^3^	Plate, pale yellow
*Z* = 4	0.47 × 0.35 × 0.12 mm

### Data collection

**Table:**

Oxford Diffraction Xcalibur Ruby Gemini diffractometer	1901 independent reflections
Radiation source: Enhance (Cu) X-ray Source	1839 reflections with *I* > 2σ(*I*)
graphite	*R*_int_ = 0.021
Detector resolution: 10.5081 pixels mm^-1^	θ_max_ = 74.0°, θ_min_ = 5.6°
ω scans	*h* = −13→19
Absorption correction: multi-scan (*CrysAlis RED*; Oxford Diffraction, 2007)	*k* = −8→8
*T*_min_ = 0.655, *T*_max_ = 1.000	*l* = −11→13
3043 measured reflections	

### Refinement

**Table:**

Refinement on *F*^2^	Secondary atom site location: difference Fourier map
Least-squares matrix: full	Hydrogen site location: inferred from neighbouring sites
*R*[*F*^2^ > 2σ(*F*^2^)] = 0.031	H-atom parameters constrained
*wR*(*F*^2^) = 0.084	*w* = 1/[σ^2^(*F*_o_^2^) + (0.060*P*)^2^ + 0.3149*P*] where *P* = (*F*_o_^2^ + 2*F*_c_^2^)/3
*S* = 1.08	(Δ/σ)_max_ = 0.001
1901 reflections	Δρ_max_ = 0.51 e Å^−^^3^
172 parameters	Δρ_min_ = −0.36 e Å^−^^3^
1 restraint	Absolute structure: Flack (1983), 472 Friedel pairs
Primary atom site location: structure-invariant direct methods	Flack parameter: 0.04 (2)

### Special details

**Table:**

Geometry. All e.s.d.'s (except the e.s.d. in the dihedral angle between two l.s. planes) are estimated using the full covariance matrix. The cell e.s.d.'s are taken into account individually in the estimation of e.s.d.'s in distances, angles and torsion angles; correlations between e.s.d.'s in cell parameters are only used when they are defined by crystal symmetry. An approximate (isotropic) treatment of cell e.s.d.'s is used for estimating e.s.d.'s involving l.s. planes.
Refinement. Refinement of *F*^2^ against ALL reflections. The weighted *R*-factor *wR* and goodness of fit *S* are based on *F*^2^, conventional *R*-factors *R* are based on *F*, with *F* set to zero for negative *F*^2^. The threshold expression of *F*^2^ > σ(*F*^2^) is used only for calculating *R*-factors(gt) *etc*. and is not relevant to the choice of reflections for refinement. *R*-factors based on *F*^2^ are statistically about twice as large as those based on *F*, and *R*- factors based on ALL data will be even larger.

### Fractional atomic coordinates and isotropic or equivalent isotropic displacement parameters (Å^2^)

**Table:**

	*x*	*y*	*z*	*U*_iso_*/*U*_eq_	
Br	0.18735 (2)	0.86647 (5)	−0.24466 (4)	0.03870 (15)	
Cl	0.01563 (7)	0.92827 (15)	0.48765 (11)	0.0500 (3)	
O1	0.36114 (15)	0.8900 (4)	0.2328 (3)	0.0367 (7)	
O2	0.3344 (2)	0.8808 (4)	0.4162 (3)	0.0452 (7)	
N1	0.31207 (17)	0.8805 (4)	0.3146 (4)	0.0272 (7)	
C1	0.3270 (2)	0.9177 (5)	−0.0882 (4)	0.0258 (7)	
C2	0.3041 (2)	0.8721 (5)	−0.2013 (4)	0.0283 (8)	
C3	0.3634 (3)	0.8310 (5)	−0.2862 (4)	0.0366 (9)	
H3A	0.3458	0.8004	−0.3626	0.044*	
C4	0.4477 (3)	0.8353 (5)	−0.2583 (5)	0.0419 (11)	
H4A	0.4890	0.8085	−0.3157	0.050*	
C5	0.4730 (3)	0.8786 (5)	−0.1466 (4)	0.0379 (10)	
H5A	0.5315	0.8804	−0.1275	0.046*	
C6	0.4135 (2)	0.9190 (5)	−0.0635 (4)	0.0315 (8)	
H6A	0.4317	0.9486	0.0127	0.038*	
C7	0.2645 (2)	0.9579 (4)	0.0073 (3)	0.0270 (7)	
H7A	0.2126	1.0118	−0.0264	0.032*	
H7B	0.2894	1.0468	0.0618	0.032*	
C8	0.2413 (2)	0.7822 (4)	0.0736 (3)	0.0244 (6)	
H8A	0.2090	0.7010	0.0215	0.029*	
H8B	0.2941	0.7186	0.0963	0.029*	
C9	0.18952 (19)	0.8201 (5)	0.1804 (3)	0.0226 (7)	
C10	0.2198 (2)	0.8680 (4)	0.2905 (3)	0.0203 (6)	
C11	0.1674 (2)	0.9012 (5)	0.3857 (3)	0.0268 (7)	
H11A	0.1905	0.9317	0.4593	0.032*	
C12	0.0819 (3)	0.8884 (5)	0.3697 (4)	0.0309 (8)	
C13	0.0477 (2)	0.8412 (4)	0.2638 (6)	0.0353 (8)	
H13A	−0.0120	0.8321	0.2543	0.042*	
C14	0.1010 (2)	0.8078 (5)	0.1726 (3)	0.0285 (7)	
H14A	0.0767	0.7745	0.1002	0.034*	

### Atomic displacement parameters (Å^2^)

**Table:**

	*U*^11^	*U*^22^	*U*^33^	*U*^12^	*U*^13^	*U*^23^
Br	0.0430 (2)	0.0377 (2)	0.0354 (2)	−0.00382 (15)	−0.0082 (2)	0.0031 (2)
Cl	0.0498 (6)	0.0514 (5)	0.0486 (6)	0.0055 (5)	0.0305 (5)	0.0064 (5)
O1	0.0212 (10)	0.0559 (15)	0.0331 (18)	0.0000 (10)	0.0030 (11)	−0.0015 (12)
O2	0.0398 (14)	0.066 (2)	0.0297 (16)	−0.0066 (13)	−0.0109 (14)	0.0040 (14)
N1	0.0258 (16)	0.0237 (15)	0.032 (2)	−0.0007 (11)	−0.0051 (13)	0.0018 (13)
C1	0.0401 (17)	0.0139 (14)	0.0234 (18)	−0.0017 (12)	0.0040 (14)	−0.0001 (13)
C2	0.0379 (19)	0.0214 (17)	0.0256 (18)	−0.0019 (13)	0.0027 (14)	0.0008 (12)
C3	0.062 (3)	0.0214 (15)	0.0261 (18)	−0.0024 (16)	0.0109 (17)	0.0031 (14)
C4	0.050 (2)	0.0329 (17)	0.042 (3)	0.0008 (15)	0.024 (2)	0.0017 (19)
C5	0.036 (2)	0.0307 (19)	0.047 (3)	−0.0015 (14)	0.0067 (18)	0.0013 (17)
C6	0.0378 (18)	0.0233 (15)	0.033 (2)	−0.0034 (14)	0.0025 (15)	0.0012 (15)
C7	0.0364 (16)	0.0208 (14)	0.0238 (17)	0.0064 (13)	0.0037 (14)	−0.0006 (12)
C8	0.0289 (16)	0.0196 (14)	0.0247 (15)	0.0023 (12)	0.0003 (12)	−0.0021 (12)
C9	0.0267 (18)	0.0149 (14)	0.0262 (19)	0.0006 (11)	0.0007 (13)	−0.0024 (14)
C10	0.0262 (16)	0.0136 (14)	0.0210 (17)	−0.0002 (11)	0.0004 (12)	0.0021 (9)
C11	0.0349 (18)	0.0198 (15)	0.0257 (19)	0.0010 (13)	0.0029 (15)	0.0018 (14)
C12	0.0359 (19)	0.0255 (17)	0.0312 (19)	0.0023 (13)	0.0181 (16)	0.0054 (14)
C13	0.0232 (14)	0.0319 (17)	0.051 (2)	−0.0028 (12)	0.003 (2)	0.005 (2)
C14	0.0261 (16)	0.0273 (16)	0.0322 (19)	−0.0031 (13)	−0.0055 (14)	−0.0010 (15)

### Geometric parameters (Å, °)

**Table:**

Br—C2	1.909 (4)	C7—C8	1.549 (4)
Cl—C12	1.739 (4)	C7—H7A	0.9900
O1—N1	1.222 (5)	C7—H7B	0.9900
O2—N1	1.222 (6)	C8—C9	1.503 (5)
N1—C10	1.485 (4)	C8—H8A	0.9900
C1—C2	1.393 (6)	C8—H8B	0.9900
C1—C6	1.393 (5)	C9—C10	1.401 (5)
C1—C7	1.507 (5)	C9—C14	1.403 (4)
C2—C3	1.388 (6)	C10—C11	1.395 (5)
C3—C4	1.368 (6)	C11—C12	1.365 (6)
C3—H3A	0.9500	C11—H11A	0.9500
C4—C5	1.385 (8)	C12—C13	1.379 (8)
C4—H4A	0.9500	C13—C14	1.368 (6)
C5—C6	1.374 (6)	C13—H13A	0.9500
C5—H5A	0.9500	C14—H14A	0.9500
C6—H6A	0.9500		
			
O1—N1—O2	123.8 (3)	H7A—C7—H7B	108.1
O1—N1—C10	118.7 (3)	C9—C8—C7	112.1 (3)
O2—N1—C10	117.5 (3)	C9—C8—H8A	109.2
C2—C1—C6	116.6 (4)	C7—C8—H8A	109.2
C2—C1—C7	124.1 (3)	C9—C8—H8B	109.2
C6—C1—C7	119.3 (3)	C7—C8—H8B	109.2
C3—C2—C1	122.5 (4)	H8A—C8—H8B	107.9
C3—C2—Br	117.5 (4)	C10—C9—C14	114.4 (3)
C1—C2—Br	120.0 (3)	C10—C9—C8	127.1 (3)
C4—C3—C2	119.0 (4)	C14—C9—C8	118.5 (3)
C4—C3—H3A	120.5	C11—C10—C9	123.7 (3)
C2—C3—H3A	120.5	C11—C10—N1	115.0 (3)
C3—C4—C5	120.3 (4)	C9—C10—N1	121.3 (3)
C3—C4—H4A	119.9	C12—C11—C10	117.9 (4)
C5—C4—H4A	119.9	C12—C11—H11A	121.1
C6—C5—C4	120.1 (4)	C10—C11—H11A	121.1
C6—C5—H5A	120.0	C11—C12—C13	121.5 (4)
C4—C5—H5A	120.0	C11—C12—Cl	118.5 (4)
C5—C6—C1	121.6 (4)	C13—C12—Cl	120.0 (3)
C5—C6—H6A	119.2	C14—C13—C12	119.0 (3)
C1—C6—H6A	119.2	C14—C13—H13A	120.5
C1—C7—C8	110.5 (3)	C12—C13—H13A	120.5
C1—C7—H7A	109.6	C13—C14—C9	123.5 (4)
C8—C7—H7A	109.6	C13—C14—H14A	118.3
C1—C7—H7B	109.6	C9—C14—H14A	118.3
C8—C7—H7B	109.6		
			
C6—C1—C2—C3	−0.4 (5)	C8—C9—C10—C11	180.0 (3)
C7—C1—C2—C3	−178.2 (3)	C14—C9—C10—N1	177.8 (3)
C6—C1—C2—Br	−179.9 (2)	C8—C9—C10—N1	−2.2 (5)
C7—C1—C2—Br	2.4 (5)	O1—N1—C10—C11	−165.3 (3)
C1—C2—C3—C4	0.0 (6)	O2—N1—C10—C11	14.7 (5)
Br—C2—C3—C4	179.4 (3)	O1—N1—C10—C9	16.7 (5)
C2—C3—C4—C5	0.5 (6)	O2—N1—C10—C9	−163.3 (3)
C3—C4—C5—C6	−0.6 (6)	C9—C10—C11—C12	−0.9 (5)
C4—C5—C6—C1	0.1 (6)	N1—C10—C11—C12	−178.8 (3)
C2—C1—C6—C5	0.4 (5)	C10—C11—C12—C13	1.1 (6)
C7—C1—C6—C5	178.3 (3)	C10—C11—C12—Cl	179.7 (3)
C2—C1—C7—C8	90.2 (4)	C11—C12—C13—C14	−0.4 (6)
C6—C1—C7—C8	−87.5 (4)	Cl—C12—C13—C14	−179.0 (3)
C1—C7—C8—C9	171.4 (3)	C12—C13—C14—C9	−0.5 (6)
C7—C8—C9—C10	−84.5 (4)	C10—C9—C14—C13	0.7 (5)
C7—C8—C9—C14	95.5 (4)	C8—C9—C14—C13	−179.3 (3)
C14—C9—C10—C11	0.0 (5)		

### Hydrogen-bond geometry (Å, °)

**Table:**

*D*—H···*A*	*D*—H	H···*A*	*D*···*A*	*D*—H···*A*
C3—H3A···O2^i^	0.95	2.62	3.479 (6)	150
C13—H13A···O1^ii^	0.95	2.60	3.421 (4)	145

Symmetry codes: (i) *x*, *y*, *z*−1; (ii) *x*−1/2, −*y*+3/2, *z*.

### Table 2 Cg···Cg π-stacking interactions, Cg1 and Cg2 are the centroids of rings C1-C6 and C9-C14. [Symmetry codes: (i) 1/2-x, -1/2+y, -1/2+z; (ii) 1/2-x, 1/2+y, -1/2+z; (iii) 1/2-x, -1/2+y,1/2+z; (iv) 1/2-x, 1/2+y,1/2+z].

**Table:**

	*Cg*X···*Cg*Y (Å)	CgX···Perp (Å)	CgY···Perp (Å)
Cg1···Cg2^i^	3.903 (2)	3.5875 (15)	-3.5830 (14)
Cg1···Cg2^ii^	3.596 (2)	-3.5429 (15)	3.5404 (14)
Cg2···Cg1^iii^	3.596 (2)	3.5404 (14)	-3.5430 (15)
Cg2···Cg1^iv^	3.903 (2)	-3.5830 (14)	3.5875 (15)
